# The Effect of Lipoic Acid Therapy on Cognitive Functioning in Patients with Alzheimer's Disease

**DOI:** 10.1155/2013/454253

**Published:** 2013-03-30

**Authors:** Antonietta Fava, Domenico Pirritano, Massimiliano Plastino, Dario Cristiano, Giovanna Puccio, Carmen Colica, Caterina Ermio, Matteo De Bartolo, Gaetano Mauro, Domenico Bosco

**Affiliations:** ^1^Department of Clinical and Experimental Medicine, University of Catanzaro, 88100 Catanzaro, Italy; ^2^Department of Neuroscience, “S. Giovanni di Dio” Hospital, 88900 Crotone, Italy; ^3^Institute of Neurological Science, National Research Council, Roccelletta di Borgia, 88021 Catanzaro, Italy; ^4^Department of Neuroscience, “S. Giovanni Paolo II” Hospital, 88046 Lamezia Terme, Catanzaro, Italy; ^5^Neurophysiology Unit, General Hospital, 87067 Rossano, Cosenza, Italy; ^6^General Medicine Unit, General Hospital, 87055 San Giovanni in Fiore, Cosenza, Italy

## Abstract

Diabetes mellitus (DM) is an important risk factor for Alzheimer's disease (AD). Most diabetic patients have insulin resistance (IR) that is associated with compensatory hyperinsulinemia, one of the mechanisms suggested for increased AD risk in patients with DM. Alpha-lipoic acid (ALA) is a disulfide molecule with antioxidant properties that has positive effects on glucose metabolism and IR. This study evaluated the effect of ALA treatment (600 mg/day) on cognitive performances in AD patients with and without DM. One hundred and twenty-six patients with AD were divided into two groups, according to DM presence (group A) or absence (group B). Cognitive functions were assessed by MMSE, Alzheimer's Disease Assessment Scale-cognitive (ADAS-Cog), Clinician's Interview-Based Impression of Severity (CIBIC), Clinical Dementia Rating (CDR), and Alzheimer's Disease Functional and Change Scale (ADFACS). IR was assessed by HOMA index. At the end of the study, MMSE scores showed a significant improvement in 43% patients of group A (26 subjects) and 23% of group B (15 subjects), compared to baseline (*P* = .001). Also ADAS-Cog, CIBIC, and ADFACS scores showed a significant improvement in group A versus group B. IR was higher in group A. Our study suggests that ALA therapy could be effective in slowing cognitive decline in patients with AD and IR.

## 1. Introduction

Alzheimer's disease (AD) is a neurological disorder characterized by profound memory loss and progressive dementia. The cause of sporadic AD remains poorly understood. In addition to genetic susceptibility genes, such as ApoE4 allele, a number of risk factors has been identified including many lifestyle and dietary choices [1]. Type 2 diabetes mellitus (T2-DM2) is an important risk factor for AD and vascular dementia [2, 3]. Recent longitudinal studies have shown that AD is related to glucose metabolism disorders [4, 5]. An explanation seems to be that vascular complications of diabetes may cause neurodegenerative disease [6]. On the other hand, in addition to its peripheral metabolic effects, insulin may also have important outcome on brain functions. A recent commentary offers two models on the relationship between T2-DM and AD: “central insulin resistance” and inflammation. Both mechanisms influence insulin sensitivity in the brain, finally leading to *β*-amyloid accumulation and, consequently, to AD [7]. Moreover, most diabetic patients have insulin resistance (IR) that is associated with compensatory hyperinsulinemia, one of the mechanisms suggested to explain the increased AD risk in diabetic patients [8, 9].

Alpha-lipoic acid (ALA) is a naturally occurring disulfide molecule with antioxidant and anti-inflammatory properties. It has positive effects on glucose metabolism and/or IR [10] and should exert positive effects in patients with AD. ALA plays many different roles in pathogenic pathways of dementia, acting as a neuroprotective agent. It increases acetylcholine production, inhibits hydroxyl radical production, and increases the process of getting rid of reactive oxygen species. By the same ways, ALA promotes downregulation of redox-sensitive inflammatory processes [11]. A decade of small epidemiological studies provides evidence about the role that ALA plays in patients with AD and related dementias [11, 12]. In these studies, the authors presented patients with mild AD showing an unusually slow progression of cognitive impairment or a stabilization of cognitive function during ALA therapy.

The aim of this study was to evaluate the effect of ALA (600 mg/day) on cognitive functions in AD patients with or without T2-DM, respectively.

## 2. Methods

### 2.1. Study Subjects

Between November 2009 and November 2011, we recruited 126 patients (75 women and 51 men) with AD according to Diagnostic and Statistical Manual of Mental Disorders, Fourth Edition (DSM-IV) criteria, associated with or/without T2-DM [13]. We enrolled patients with a MMSE score raging between 12 and 26. In accordance with the official standards of the 1964 declaration of Helsinki, local laws and regulations, for each patient, informed consent was obtained by care givers. Differential diagnoses have been made according to the National Institute of Neurological and Communicative Disorders and Stroke—Alzheimer's disease and Related Disorders Association Internationale pour la Recherché et l'Enseignement en Neurosciences criteria for vascular dementia [14]. We excluded subjects with (a) history of psychiatric disease (depression and/or antidepressant intake) and/or behaviour disorders; (b) tumour and/or vascular disease; (c) toxic or pharmacological exposure, alcohol overuse, and coexisting medical conditions associated with cognitive impairment (i.e., dysthyroidism, human immunodeficiency virus); (d) abnormal results on blood tests, including electrolytes, renal and hepatic function, B12 and folic levels, serum protein electrophoresis with serum immunofixation, and autoimmune and infective screening; (e) hydrocephalus; and (f) extrapyramidal disorders.

Enrolled patients were divided into two groups as follows, according to the presence of T2-DM:

 group A—AD patients with T2-DM (61 patients);
 group B—AD patients without T2-DM (65 patients).

### 2.2. Clinical Evaluation

After a first-level checkup, carried out by a neurologist and/or a geriatrician expert in neurodegenerative disease and by an endocrinologist, patients underwent neuroimaging studies (CT scan or MRI of the brain) and neuropsychological assessment. Recorded medical data included anamnestic (health and behaviour status, disease duration, antipsychotic use) and demographic data (age, gender), body mass index (BMI), waist circumference (WC), glucose and insulin fasting values, triglycerides, and blood lipid profile. Cardiovascular disease (heart disease and hypertension) and smoking habit were defined by self-reports. History of T2-DM was ascertained by patient self-reports or antidiabetic medication use. IR was calculated by homeostasis model assessment (HOMA) formula [15]. We defined DM as documented insulin or oral hypoglycaemic drug use or fasting plasma glucose level above 200 mg/dL (11.1 mmol/dL) [16].

### 2.3. Neuropsychological Assessment

All patients underwent a general cognitive screening with Mini-Mental State Examination (MMSE) [17]. We considered as significant an improvement/worsening of two points or more at the MMSE; a variation of one point or less was considered as not significant. Depression was assessed with Montgomery Asberg Depression Rating Scale (MADRS) [18]. Patient evaluation included Alzheimer's Disease Assessment Scale, cognitive subscale (ADAS-Cog) [19], and the Clinician's Interview-Based Impression of Change-Plus version (CIBIC-plus) [20]. Moreover, we used global Clinical Dementia Rating (CDR), the CDR sum of boxes (the sum of individual CDR domain scores) [21], and Alzheimer's Disease Functional and Change Scale (ADFACS) [22]. ADFACS provides a measure of instrumental and basic activities of daily living. Patients score's were compared with normal values according to age and educational level.

### 2.4. Study Design

This is a prospective, open label, parallel-group study, performed from November 2009 to November 2011, in an outpatient setting, in two towns of south Italy (Crotone and Lamezia Terme, Calabria). All patients were assigned to receive ALA (600 mg/day) in combination with antidementia treatment. Study duration was 16 months and consisted of three consecutive periods. At study entry (V1), all patients' care givers gave an informed consent, and each patient underwent neurological, endocrinological, and neuropsychological evaluation. Clinical and neuropsychological assessments were also performed at month 8 (V2) and at the end of the study (V3; month 16). The neuropsychological assessments were performed by trained neuropsychologists blind to group stratification. Fasting serum glucose, insulin and triglycerides values, lipid profile, smoking habits, and antidiabetic and antipsychotic medications were also assessed at V1 and V3. For the entire study duration, dementia treatments (cholinesterase inhibitors (ChE) (donepezil, rivastigmine, and galantamine) and the N-methyl-D-aspartate (NMDA) receptor antagonist memantine) were kept fixed for each patient (donepezil between 5 and 10 mg/day, rivastigmine between 6 and 12 mg/day, galantamine between 8 and 16 mg/day, and memantine between 10 and 20 mg/day). ALA therapy began about 3 months after antidementia treatment. Care givers were responsible for all drug intake and for patients' adherence to therapy.

### 2.5. Data Analysis

Data were expressed as mean ± standard deviation (SD). In the Intention-to-Treat (ITT) population, all patients who received at least one dose of ALA (600 mg/day) were included. No confirmatory statistical testing was performed. Results were summarized using descriptive statistic. Baseline and demographic characteristics were summarized for all enrolled patients. An ANOVA test for independent samples was performed to compare mean values. A *χ* ^2^ test was performed to compare prevalence data. Finally, logistic regression was used to assess and allow for discrepancies among clinical characteristics in the group comparisons and to assess relative significance of potential aetiology variables. Besides, we included variables that are usually associated with higher risk of dementia (i.e., age, sex, glycaemia, education level, disease duration, smoking habit, etc.). 

MMSE, ADAS-Cog, CDR, and ADFACS scores were summarized using means for patients' scores at V1, V2, and V3. CGI-C and MMSE scores were assessed in terms of proportions of patients showing improvement, worsening, or no change from previous visit. In all cases, *P* value of .05 was considered statistically significant. Statistical analysis was performed using SPSS 12.0.

## 3. Results

### 3.1. Outcome and Adverse Events

**Figure 1:**
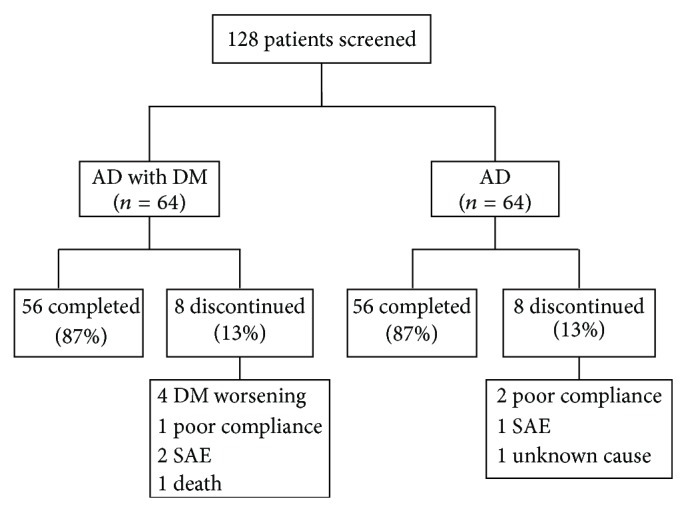
Patients flow-chart. Sixteen patients dropped out of trial: 11 (15%) in group A and 5 (7%) in group B for marked diabetes mellitus (DM) worsening in 6 (all in group A); poor compliance in 3 (1 in group A and 2 in group B); severe adverse events (SAEs) in 3 (2 in group A and 1 in group B); unknown cause in 2 (in group B), and 1 death (a stroke, in group A).

One hundred and twenty-six patients (88,7% of the ITT/population) completed the study: 61 in group A and 65 in group B. Sixteen patients dropped out prematurely (11 in group A and 5 in group B): marked T2-DM worsening in 6 (all in group A); poor compliance in 4 (2 in group A and 2 in group B); severe adverse events (SAEs) in 3 (2 in group A and 1 in group B); unknown cause in 2 (in group B); and one death (a stroke, in group A) occurred during the study and judged unrelated to treatment. ALA was well tolerated in all patients. Forty-four percent (27/61) of patients in group A and 41% (27/65) in group B showed adverse events (AEs). AEs included muscle cramps, gastrointestinal symptoms, and sleep disturbances in both groups. Among dropped out patients, two had severe sleepiness and one profuse diarrhoea. All SAEs were judged to be related to donezepil treatment. Thus, only 126 patients (61 group A and 65 group B) were included in the analysis (Figure 1).

### 3.2. Demographics

Table 1 summarizes demographic and clinical characteristics of patients. Mean age was 72 ± 6.8 years in group A and 74.2 ± 5.7 years in group B. The percentage of man in group A was significantly higher than group B (36% versus 44.6%; *P* = .05). Consequently, any statistical comparison between the groups took gender into account. Mean AD duration was similar in both groups. Educational level was 10.6 ± 4.5 years in group A and 11.7 ± 5.4 years in group B, respectively. During the study, all patients received medications for dementia. Smoking use was higher in group B, but after adjusting for sex the difference was not significant (*P* = .09). Sixty-eight percent (*n* = 86) of patients had one or more concomitant pathologies, 56 (44%) in group A and 30 (24%) in group B: 38 were past and 48 current pathologies. Hypertension, ischemic heart disease, and hypercholesterolemia were among the most frequent concomitant pathologies. 

### 3.3. Metabolic and Clinical Features

At presentation (V1), mean BMI, WC, serum lipid, and triglycerides were similar in groups A and B. Mean HOMA value was 10.2 ± 4.2 in group A and 1.6 ± 0.8 in group B, respectively (*P* = .001). At visit V3: HOMA index value between groups was lower compared to baseline, but remained significantly higher in group A (*P* = .03); the other metabolic parameters (serum lipid and triglycerides) did not show significant differences between the two groups. Besides, at the beginning of the study, 9 patients in group A were taking insulin therapy alone or in combination with oral antidiabetic agents, the other patients were taking only oral antidiabetic drugs. At the end of the study, 5 patients required the administration of insulin, while the 12 patients increased oral antidiabetic dosage (Table 2).

### 3.4. Neuropsychological Findings

Neuropsychological assessments were performed at times V1, V2, and V3. Patients from ITT population were included in neuropsychological analysis if they had completed the study and had a final assessment within 7 days after 16 months. Comparison of neuropsychological differences between the groups is summarized in Table 3. MMSE score demonstrated that overall dementia levels improved in group A compared with group B at any evaluation time, reaching a statistical significance at V2 (*P* = .001) and V3 (*P* = .003). For the primary outcome, patients in ITT population had a significant improvement in MMSE scores from the baseline at the end of the study (V3) in 43% in group A and 23% in group B (*P* = .003). Thirty-three percent of patients in group A and 40% in group B showed significant worsening from baseline (*P* = .05). Besides, patients in group A demonstrated significant improvements versus group B on ADAS-cog, at all evaluation times. Global function improvement, assessed by CIBIC-plus, was observed in a greater proportion of group A patients compared to group B, at V3 (*P* = .001). Significant benefits were not observed on CDR-SB. After an initial improvement, group A declined below baseline on CDR-SB at the end of the study (*P* = .39). Analysis of ADFACS total scores demonstrated significant functional benefits in group A. ADFACS scores remained close to the baseline values in group A, whereas group B declined. Separate analysis of ADFACS instrumental ADL (IADL) items also demonstrated significant differences between groups.

## 4. Discussion

In this prospective study, we evaluated the effects of alpha-lipoic acid (600 mg/day) in 126 patients with mild-to-moderate AD with or without T2-DM, respectively. We hypothesize that ALA therapy, in combination with antidementia drugs, may have an effect on cognitive functions and could slow the progression of dementia in AD patients. The results of our study in a real life, naturalist clinical setting indicate that a large percentage of AD patients with T2-DM (group A) could benefit from ALA therapy; in fact, we documented a significant slowing in cognitive decline, at 8- and 16-month followup in this group of patients. Among AD patients with T2-DM around 43% improved over 16 months on MMSE-scores; moreover, daily living items showed a significant improvement in most of patients in group A versus group B. Besides, 47% of patients in group A showed significant improvement on the CIBIC-plus rating after 16 months (*P* = .001).

We did not observe a significant benefit on CDR-SB. This may be due to some intrinsic limitations of this scale, including the relative insensitivity as a measure of change in pharmacological studies [23]. Complex molecular mechanisms, referring to insulin and/or insulin-like growth factor-1 (IGF-1) signalling, could link DM to AD [24]. In fact, there is evidence that altered insulin and/or IGF-1 signalling to brain cells may be responsible for beta-amyloid accumulation in AD [25]. Moreover, most diabetic patients have IR that is associated with compensatory hyperinsulinemia, one of the mechanisms suggested to explain the increased AD risk in diabetic patients [8, 26]. Subsequent investigations demonstrated reduced blood glucose levels and increased insulin levels in patients with late onset AD compared to aged controls or to patients with vascular dementia. Although the authors concluded that these findings did not support an association between diabetes and AD [27], the same data were reinterpreted as an increased prevalence of IR in AD. Further studies demonstrated that the administration of insulin significantly improved memory performance in AD patients [9, 28].

ALA, a biological antioxidant and natural cofactor of mitochondrial dehydrogenase complexes, is considered to be safe and efficacious for treatment of diabetic polyneuropathy [29]. ALA, which is synthesized by liver, exists as two different enantiomers: the biologically active- (R-) isomer, and the (S)-isomer, that is found in biological tissue in very small amounts. In vitro studies have provided evidence that R-(+)-ALA can specifically activate two important molecules of the insulin signalling pathway-insulin receptor substrate-1 (IRS-1) protein and phosphatidylinositol 3-kinase, with subsequent enhancement of glucose uptake, via the glucose transporter system in skeletal muscle and adipocytes [30]. ALA has been shown to improve insulin sensitivity in cell cultures of skeletal muscles [31] and in animal model of T2-DM [32]. The effect of ALA therapy on cognitive decline is controversial [10, 33]. A longitudinal study in dogs showed that a diet enriched with a broad spectrum of antioxidants, along with ALA, induced rapid improvements in landmark performance and prevent age-related cognitive decline in old dogs [34]. Conversely, Christie et al. reported no effects of short-term supplementation with ALA and acetyl-L-carnitine on cognition in aged dogs [33]. In one small open-label study, ALA (600 mg/daily) was given to nine patients with probable AD. Treatment with ALA led to a stabilization of cognitive functions, demonstrated by constant scores in two neuropsychological tests for a period of 12 months [12]. These authors have subsequently extended the analysis to 43 patients with an observation period of 48 months. Cognitive progression appeared dramatically slower compared to untreated patients or patients on choline-esterase inhibitors, in the second year of long-term studies [35]. Moreover, Siedlak et al., in a recent work, suggest that supplementation with ALA is insufficient to improve cognitive performance in aged or AD models [36].

In the present study, we showed no significant difference in BMI, WC, serum lipids, triglyceridemia, and smoking use between groups. Nevertheless, hypertension, ischemic heart disease, and HOMA value were significantly higher in AD with T2-DM group; however, in our study, after adjustment for these factors, the improvement of cognitive performances remained significantly greater in the group of patients with concomitant DM at the end of the study (16-months). We suppose that ALA supplementation could have an independent effect on cognitive performance. This association could be explained through several mechanisms: (i) increased acetylcholine (Ach) production by activation of choline-acetyltransferase; (ii) increased glucose uptake, supplying more acetyl-CoA for the production of Ach; (iii) inhibiting the formation of hydroxyl radicals; (iv) scavenging reactive oxygen species (ROS), downregulating inflammatory processes; (v) scavenging lipid peroxidation products; and (vi) inducing enzymes of glutathione synthesis [37, 38]. We assumed an additional pathogenetic mechanism, suggesting that the benefit on cognitive function in our patients could be related to IR improvement due to ALA. This is confirmed by a marked IR value reduction at the end of the study (IR: T0–T16; 10.2 ± 4.2–4.8 ± 2.3).

Our study has some limitations: diagnosis of AD was performed only by clinical criteria; nevertheless, the clinical evaluation was accurate and complete [39]. We did not check our patients for biochemical markers that relate to amyloid or oxidative stress. Besides, the effect of concomitant insulin therapy on cognitive performance was not quantified [9]; however, the number of patients taking insulin (*n* = 14) was small compared to the number of all examined patients. Finally, our data support a causal link between IR and AD pathogenesis. Moreover, it is important to point that this is an observational study, and definitive conclusions about the real efficacy of ALA therapy in AD cannot be made at this time. Further studies on larger samples should be warranted.

## Figures and Tables

**Table 1:** Demographic and clinical features of 128 patients with Alzheimer's disease with/or without diabetes mellitus (T2-DM).

	AD with T2-DM (group A) *n* = 61	AD without T2-DM (group B) *n* = 65	*P*
Age; years, *M *(SD)	72 (6.8)	74.2 (5.7)	.32
Sex (M/F)	22/39	29/36	.05
MMSE score; *M *(SD)	20.7 (4.4)	21.8 (4.9)	.32
MADRS score	15.8	16.3	.42
Smokers; *n*, %	12; 20	18; 28	.09
Educational level; years, *M *(SD)	10.6 (4.5)	11.7 (5.4)	.21
Disease duration; years, *M *(SD):			
(a) Alzheimer disease	5.3 (2.1)	4.9 (2.8)	.12
(b) Diabetes Mellitus	7.8 (3.7)	//	//
Anti-Alzheimer drugs: *n*; %			
(a) Donezepil	21; 34	19; 29	.23
(b) Rivastigmine	28; 46	26; 40	.18
(c) Galantamine	3; 4	3; 4.6	.24
(d) Memantine	4; 6	3; 4.6	.43
Antipsychotic use: *n*; %			
(a) Total	21	27	.09
(b) Atypical antipsychotic	10; 16	15; 23	.06
(c) Typical antipsychotic	11; 12	11; 18	.12
Concomitant diseases			
(a) Total pathologies	56	30	.001
(b) Past	25	13	.03
(c) Current	31	17	.02
Current Pathologies			
(a) Hypertension	20	10	.02
(b) Hypercholesterolemia	11	7	.08
Regresses pathologies			
(a) Ischemic heart disease	13	9	.05
(b) Others pathologies	12	4	.02

MMSE: Mini-Mental State Examination; MADRS: Montgomery Asberg Depression Rating Scale. Values are expressed as mean (SD) unless otherwise indicated. *P* value of 0.05 was considered statistically significant.

**Table 2:** Changes from baseline in clinical and metabolic features in patients of groups A and B at baseline (V1) and after 16 months of followup (V3) both groups.

	Baseline (0 month)		*P*	V3 (16 months)		*P*
	Group A *M* (SD)	Group B		Group A *M *(SD)	Group B
Adjusted for sex, hypercholesterolemia, ischemic heart disease, and hypertension						

Fasting glycaemia (mg/dL)	129 (10)	66 (9.3)	.001	126 (11.4)	70 (8.6)	.02
Fasting insulinemia (UI/mL)	33 (8.7)	7.2 (2.2)	.001	15 (3.3)	6.1 (1.9)	.02
HOMA index	10.2 (4.2)	1.6 (0.8)	.001	4.8 (2.3)	1.1 (0.6)	.03
Triglycerides (mg/dL)	166 (35.8)	157 (33)	.23	159 (36)	155 (34)	.18
Serum lipid (mg/dL)	138.7 (24)	132.7 (32)	.18	134.7 (25.9)	135.6 (33)	.23
BMI	24.1 (2.6)	22.7 (3.1)	.09	24 (2.8)	23.8 (2.7)	.58
Waist circumference (cm)	81.5 (1.7)	80.7 (2.1)	.21	82 (2.1)	81.4 (2.9)	.22
Anti diabetic medication; *n *						
(a) Hypoglicemic drugs	50	//	//	46	//	//
(b) Insuline + hypoglicemic drugs	3			3		
(c) Insulin	6			11		

Body mass index: BMI [Weight (kg)/height^2^(h^2^)]; Insulin resistance (IR) was calculated by the homeostasis model assessment (HOMA) formula. HOMA index: basal glucose plasma (mg/dL) × basal insulin plasma (UI/mL)/405; differences between the proportions with insulin resistance ≥ 2.7 on the HOMA formula. Values are expressed as mean (SD) unless otherwise indicated. *P* value of 0.05 was considered statistically significant, after adjusting for confounding variables (sex, serum lipid, triglycerides, BMI, WC, ischemic heart disease, and hypertension).

**Table 3:** Changes from baseline in neuropsychological measures after 8 months (V2) and 16 months of followup (V3) in patients of groups A and B.

Change in Score from Baseline						
	8 months			16 months		
	AD with T2-DM Group A	AD without T2-DM Group B	*P *	AD with T2-DM Group A	AD without T2-DM Group B	*P*
	*n* = 61	*n* = 65		*n* = 61	*n* = 65	
MMSE	−031 ± 1.2	0.64 ± 1.19	.002	−0.85 ± 1.4	−1.42 ± 1.9	.001
MMSE (*n*; %)						
(i) Improvement	26; 43	13; 20	.001	26; 43	15; 23	.003
(ii) Unchanged	12; 38	22; 34	.003	15; 24	24; 37	.001
(iii) Worsening	12; 20	30; 46	.001	20; 33	26; 40	.05
ADAS-Cog original; *M* (SD)	0.17 (3.4)	0.95 (3.8)	.001	2.2 (4.9)	2.9 (5.4)	.05
ADAS-Cog modified; *M* (SD)	−0.59 (4.7)	0.27 (4.9)	.001	1.9 (5.7)	2.5 (6.9)	.002
CIBIC-Plus Category (*n*; %)						
(i) Improvement	24; 39	20; 31	.05	29; 47	21; 32	.001
(ii) Unchanged	16; 26	21; 32	.05	11; 18	15; 23	.09
(iii) Worsening	21; 34	24; 37	.22	21; 34	29; 45	.08
CDR sum; *M* (SD)	0.50 (1.2)	0.75 (1.46)	.09	1.5 (1.7)	1.67 (2.2)	.39
ADFACS; *M* (SD)	0.53 (0.38)	1.56 (0.42)	.002	0.37 (0.4)	1.6 (0.4)	.002
IADL; *M* (SD)	0.87 (0.32)	0.13 (0.27)	.002	0.9 (0.37)	0.03 (0.3)	.001

ADAS-Cog: Alzheimer's Disease Assessment Scale, cognitive subscale; CIBIS: Clinician's Interview-Based Impression of Severity; CDR: Clinical Dementia rating; ADFACS: Alzheimer's Disease Functional and Change Scale; ADL: Activities of Daily Living. Values are expressed as mean standard deviation (SD) unless otherwise indicated.

*P* Value of 0.05 was considered statistically significant, after adjusting for confounding variables (sex, serum lipid, triglycerides, BMI, WC, HOMA value, ischemic heart disease, and hypertension).
